# Global research status and frontiers on ferroptosis in hepatocellular carcinoma: a comprehensive bibliometric and visualized analysis

**DOI:** 10.3389/fimmu.2025.1549600

**Published:** 2025-05-02

**Authors:** Jiayu Zhu, Shengping Luo, Fei Yu, Kewei Sun

**Affiliations:** ^1^ The First Clinical College of Chinese Medicine, Hunan University of Chinese Medicine, Changsha, China; ^2^ School of Integrated Chinese and Western Medicine, Hunan University of Chinese Medicine, Changsha, China; ^3^ Department of Liver Disease, The First Hospital of Hunan University of Chinese Medicine, Changsha, China

**Keywords:** ferroptosis, hepatocellular carcinoma, bibliometric analysis, visualized, visualization analysis, VOSviewer, CiteSpace

## Abstract

**Objective:**

The purpose of this study was to analyze the research hotspots and future trends in the field of ferroptosis in hepatocellular carcinoma in the past 10 years by using bibliometrics and visualization software, and to provide reference for future research directions in this field.

**Methods:**

The Web of Science database was searched from January 1, 2012, to October 30, 2024, and the annual publication volume, countries, institutions, journals, authors, references, keywords, and other information in this field were analyzed by bibliometrics, VOSviewer, and CiteSpace.

**Results:**

A total of 645 English articles from 729 institutions in 32 countries were included in this study, with 4545 authors published in 261 journals. In the past three years, 518 articles were published, accounting for 80.3%. China has the most publications, followed by the United States. Frontiers in Oncology had the highest number of papers (n=26), while Cell had the highest number of citations (n=1206). The current research mainly focuses on two aspects: one is the study of the mechanism of ferroptosis to explore new therapeutic targets, and the other is the exploration of therapeutic methods, such as photodynamic therapy and nanomaterials, in order to inhibit the proliferation of tumor cells, reduce drug resistance, and enhance the efficacy by regulating ferroptosis, which may become a future development trend.

**Conclusion:**

In recent years, there have been increasing studies on the association between ferroptosis and hepatocellular carcinoma. This is the first comprehensive bibliometric study, which provides a reliable reference for future research in this field and promotes its further development.

## 1 Introduction

According to data from the Global Cancer Observatory 2022, liver cancer was the 6th most common cancer in incidence and the 3rd mortality rate, with a whopping 88% (1). It is estimated that, by 2025, >1 million individuals will be affected by liver cancer annually. There are three types of liver cancer, among which hepatocellular carcinoma (HCC) is the most common, accounting for 75% ~ 85%. At present, the common treatment methods for HCC include hepatectomy, liver transplantation, ablation, endovascular intervention, radiotherapy, immunotherapy, targeted therapy, etc., and surgery is still the curative treatment for HCC (2). Since most patients have no significant symptoms in the early stage, they are diagnosed at an advanced stage at the first visit, and there is no condition for surgery, and the treatment methods are limited. Therefore, the incidence of HCC is still on the rise. The five-year relative survival rate for HCC patients is only about 18% (3). Consequently, it is urgent to study the pathogenesis of HCC, explore specific targets in the early stage of HCC, and find new drugs.

Ferroptosis is a novel non-apoptotic form of cell death, first proposed by Dixon in 2012 (4). Its essence is lipid peroxidation. The main reason is that excess Fe^2+^ accumulates in the cell to form an unstable iron pool, generates reactive oxygen species (ROS) represented by hydroxyl radicals, and finally causes cell function loss or death due to membrane lipid peroxidation. Since the concept of ferroptosis was proposed, it has attracted much attention, and more and more related studies have been conducted. Ferroptosis has been investigated in a variety of cancers, including colorectal cancer (5), breast cancer (6), head and neck cancer (7), gastric cancer (8), lung cancer (9), urological malignancies (10), and so on. The vulnerability of tumor cells to ferroptosis is closely related to their dependence on iron for growth and reproduction, reflecting the long-term effects of iron in tumor progression (11). New research has found that ferroptosis can modulate the function of T cells, allowing them to exert immune roles in recognizing and eliminating cancer cells (12). Ferroptosis is involved in the physiological and pathological changes of tumors by regulating multiple pathways (13, 14). At the same time, chemotherapy drugs, targeted therapy, and radiotherapy for HCC can directly or indirectly activate ferroptosis and enhance cytotoxicity, thereby increasing the sensitivity of HCC cells to different treatments (15). Therefore, it is essential to fully understand the correlation between ferroptosis and the pathogenesis and treatment strategies of liver cancer.

In the past decade, with the rapid development of the medical field and the deepening of related research, the literatures exploring the correlation between HCC and ferroptosis has increased yearly, but there are no relevant bibliometric studies. Bibliometrics, a statistical method proposed by Alan Pritchard in 1969, is crucial for evaluating research achievements and identifying high-impact papers in a specific field (16). By analyzing information from published literature, it provides a comprehensive understanding of the development process of a field and helps to infer the current research status and hotspots within that domain (17). It has been widely used in medical fields such as oncology (18), encephalopathy (19), rheumatology, immunology (20) and gynecology (21). This article systematically analyzes the research on ferroptosis in HCC through bibliometrics and visualization, analyzes the correlation between the two from the perspectives of countries, institutions, journals, authors, references and keywords, identifies the main contributors, institutions, countries and current research priorities, and looks for development trends, in order to provide research directions and ideas for the further development of this field.

## 2 Materials and methods

### 2.1 Data collection

**Figure 1:**
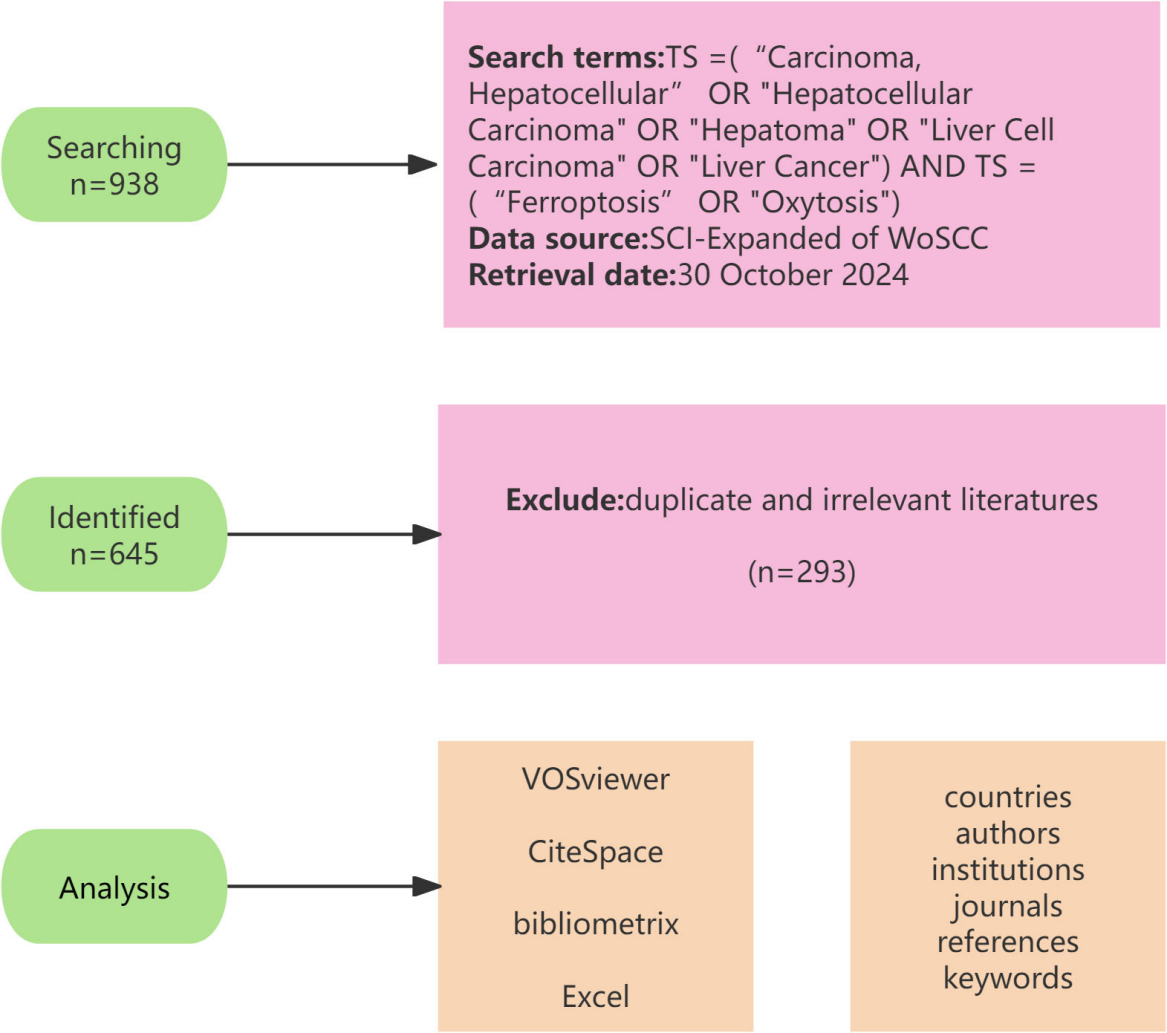
The detailed process of literature screening.

We systematically searched for articles in the Science Citation Index Expanded (SCI-Expanded) of the Web of Science Core Collection (WoSCC). Since the concept of ferroptosis was introduced in 2012, we have set the search period to span from 1 January 2012 to 30 October 2024. To ensure the accuracy and consistency of the searches, we downloaded all of the literature and related data on 30 October 2024. It was important to note that the search terms “hepatocellular carcinoma” and “ferroptosis” correspond to their respective entries in the Medical Subject Headings (MeSH) thesaurus used in the search query. The search terms included: TS = (“Carcinoma, Hepatocellular” OR “Hepatocellular Carcinoma” OR “Hepatoma” OR “Liver Cell Carcinoma” OR “Liver Cancer”) AND TS = (“Ferroptosis” OR “Oxytosis”). Initially, 938 documents were retrieved; after excluding duplicate and irrelevant literatures, we finally obtained 645 literatures (540 articles,105 reviews) that met the criteria for inclusion in the analysis. Before analyzing the data, standardize the data processing, including the normalization of countries, names, institutions, journal abbreviations, and various expressions of the same concept. The retrieval process is shown in **Figure 1**.

#### 2.1.1 Inclusion Criteria

1. Literatures related to ferroptosis and Hepatocellular Carcinoma’s disease;
2. Published in English;
3. With full bibliographic information encompassing title, country/corresponding author affiliation, authors, keywords, and publication source;
4. The literature encompassed various types, such as clinical trials, laboratory experiments, animal studies, analyses from public databases, reviews, and so on.

#### 2.1.2 Exclusion Criteria

1. Duplicated literatures;
2. Literatures with low relevance to the topic;
3. Retracted literatures.

### 2.2 Analysis tools and methods

We exported and uploaded the retrieved literature that met the requirements in a plain text format to VOSviewer 1.6.19, CiteSpace 6.2.4 Advanced, R Package Bibliometrix 4.3.1, and WPS Office Excel for bibliometric and visual analysis. VOSviewer and Bibliometrix are used to analyze contributions and collaborations by countries, authors, institutions, journals, references, and keywords. CiteSpace analyzes journals’ double map overlays, timeline plots, keyword clustering, and strongest citation burst graphs. Excel is employed for relevant data analysis and chart creation. We have chosen the right software to analyze different aspects of our research results to achieve the best presentation.

## 3 Results

### 3.1 Analysis of the publications

**Figure 2:**
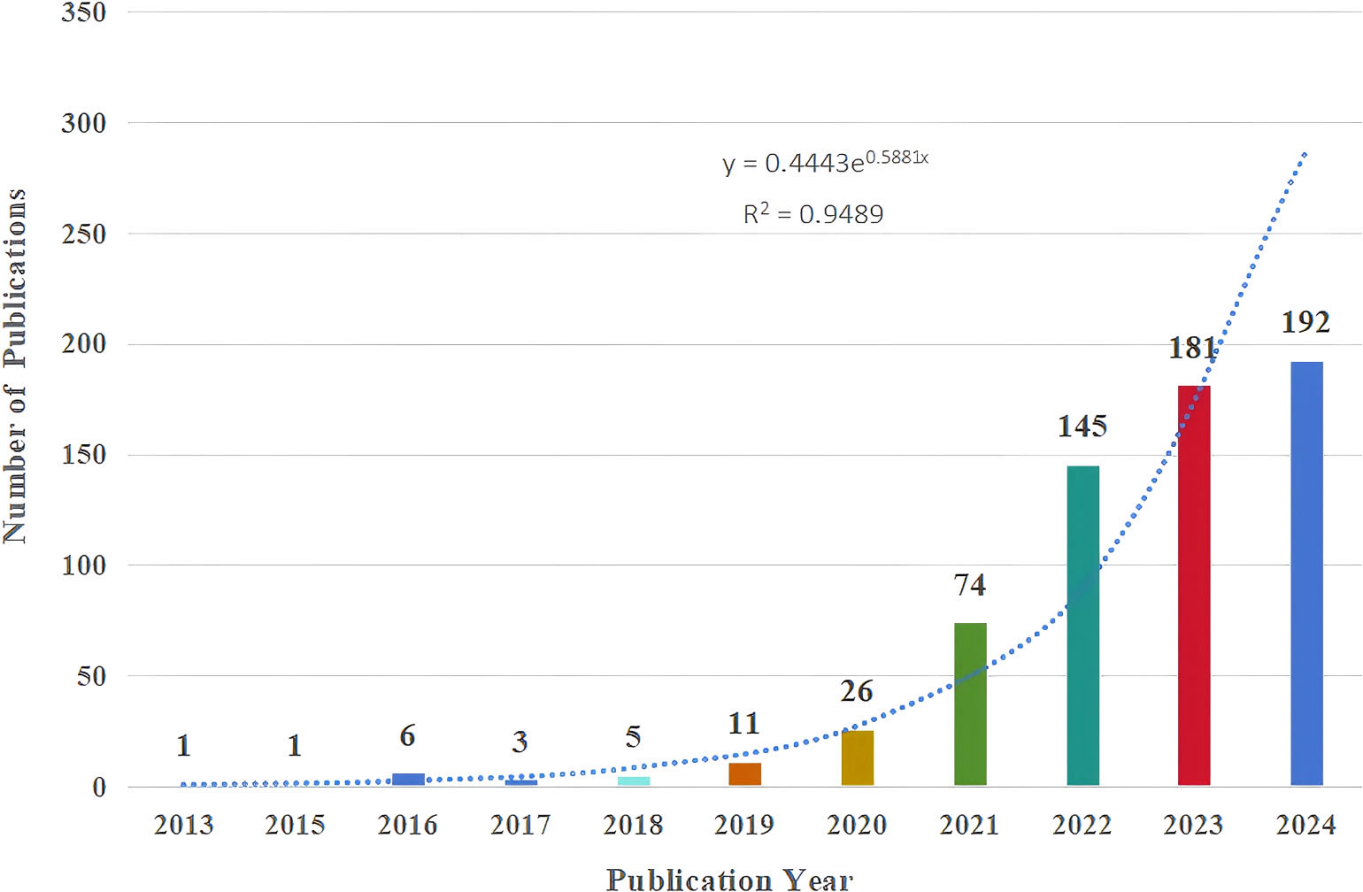
Annual number and growth trends of publications.

We further analyzed the included 645 publications. As shown in **Figure 2**, from 2013 to 2018, the number of relevant papers stabilized at 1-6. However, a significant surge was observed from 2019 onwards, with 11 publications in 2019, a significant annual publication surge to 145 in 2022, and a continuation of the upward trend in 2023, with the number of publications reaching 181. The correlation between annual publications and year is significant with a correlation coefficient of R^2^ = 0.9489, and in order to show a more complete picture of the trend of growth in annual publications, the exponential equation y = 0.4443e^0.5881x^ is used, whereby the number of publications is expected to exceed 300 by the end of 2024. Based on this curve, the number of annual publications is anticipated to rise steadily, indicating a growing interest in iron death in HCC. People are exploring the prevention and treatment strategies of HCC through the mechanism of ferroptosis to provide new ideas for future development.

### 3.2 Country analysis

**Figure 3:**
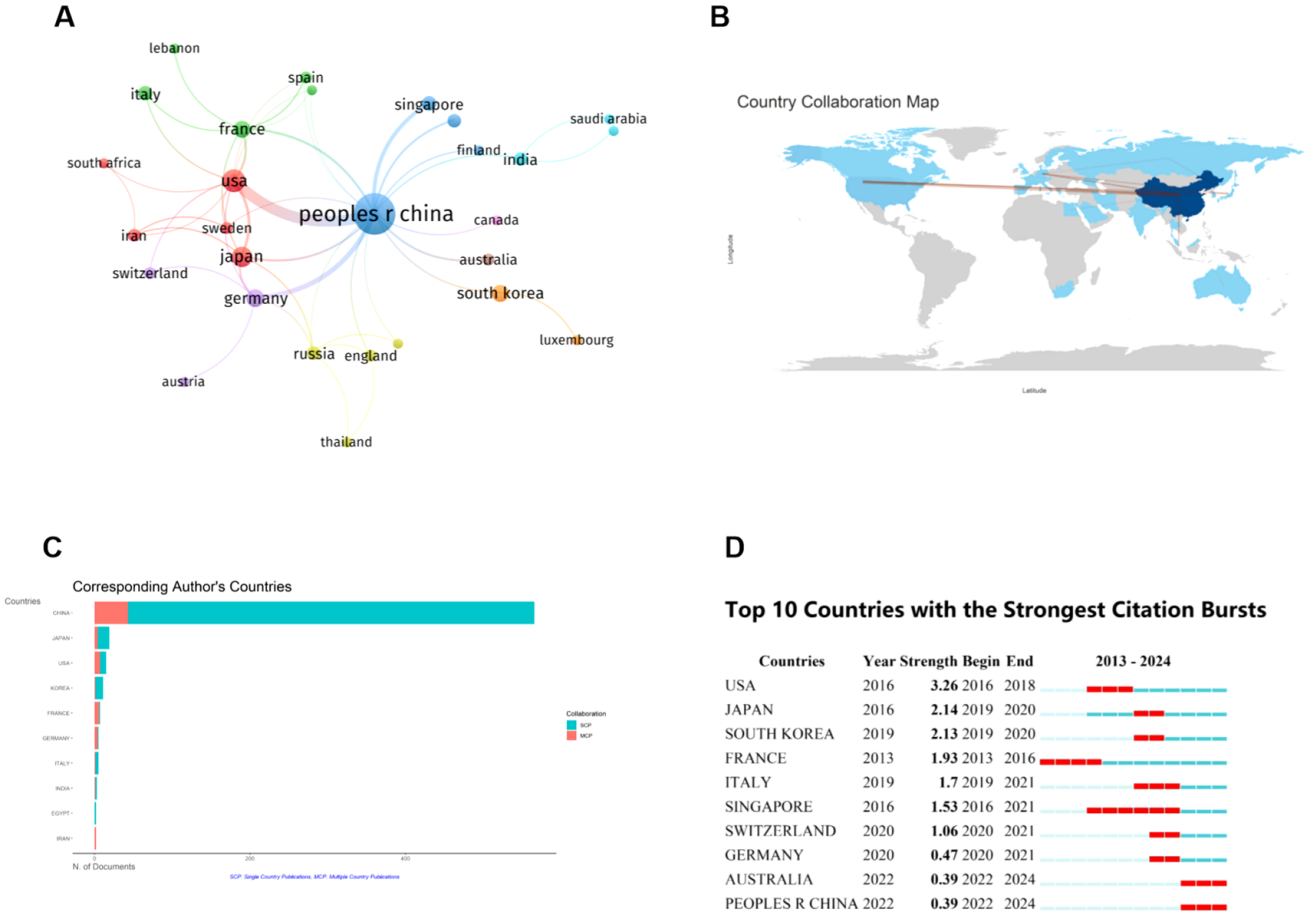
Contributions of various countries to the research of ferroptosis in HCC. **(A)** Map of National Cooperation Contributions. The circles correspond to the number of publications from each country, and the lines represent their collaborations. **(B)** World map of countries’ cooperation. **(C)** The 10 countries with the most corresponding authors. **(D)** Top 10 Countries with the Strongest Citation bursts. The red line shows the time frame during bursts were discovered, while the blue line shows the time interval. From: VOSViewer, bibliometrix.

At present, 32 countries throughout the world have participated in research related to the ferroptosis of HCC, and the top 10 countries with the greatest number of publications are listed in **Table 1**. China is the most productive country (n=575), and it is also the most cited country (n=16118), followed by the United States (n=43) and Japan (n=25). **Figures 3A, B** illustrate the collaborative relationships between countries in ferroptosis and HCC research. The size of nodes in the graph corresponds to the quantity of publications released by each country, while the thickness of connecting lines indicates the strength of collaborative relationships between countries. China’s cooperative relationships with 17 countries suggest its crucial role in international collaborations and highlight its substantial research contribution to the global landscape of ferroptosis in HCC. Among them, China and the United States have the closest cooperation, with a link strength of 24.75. It shows that Chinese and American scholars have made significant contributions to research in this field and have carried out the most cooperation. As shown in **Figure 3C**, a single national publication (SCP) indicates that all authors of an article are from the same country. In contrast, Multiple Country Publications (MCP) suggest that the authors of an article are from more than one country. Presumably, China and the USA have the largest number of foreign co-authors in this field, while intra-Chinese cooperation still accounts for a large proportion.

**Table 1:** Top 10 most productive countries.

Ranking	Countries	Documents	Citations	Average Citations/Publication
1	China	575	16118	28.03
2	USA	43	6331	147.23
3	Japan	25	645	25.8
4	Germany	14	522	37.29
5	South Korea	12	453	37.75
6	France	11	1206	109.64
7	Italy	6	295	49.17
8	Singapore	6	634	105.67
9	Russia	4	184	46
10	India	4	13	3.25

The citation explosion in various countries demonstrates the dynamic trajectory of the field of study, as well as the time frame of the surge in citation volume. **Figure 3D** presents the citation bursts for the top 10 countries. The red line depicted on the graph represents the magnitude of the citation bursts for each of the prominent countries. During the period from 2013 to 2024, USA experienced a notable surge in publications (strength = 3.26), closely followed by Japan (strength = 2.14) and South Korea (strength = 2.13). This suggests that paying attention to U.S. dynamics can help us identify new trends and research hotspots in specific research areas.

### 3.3 Institution analysis

**Figure 4:**
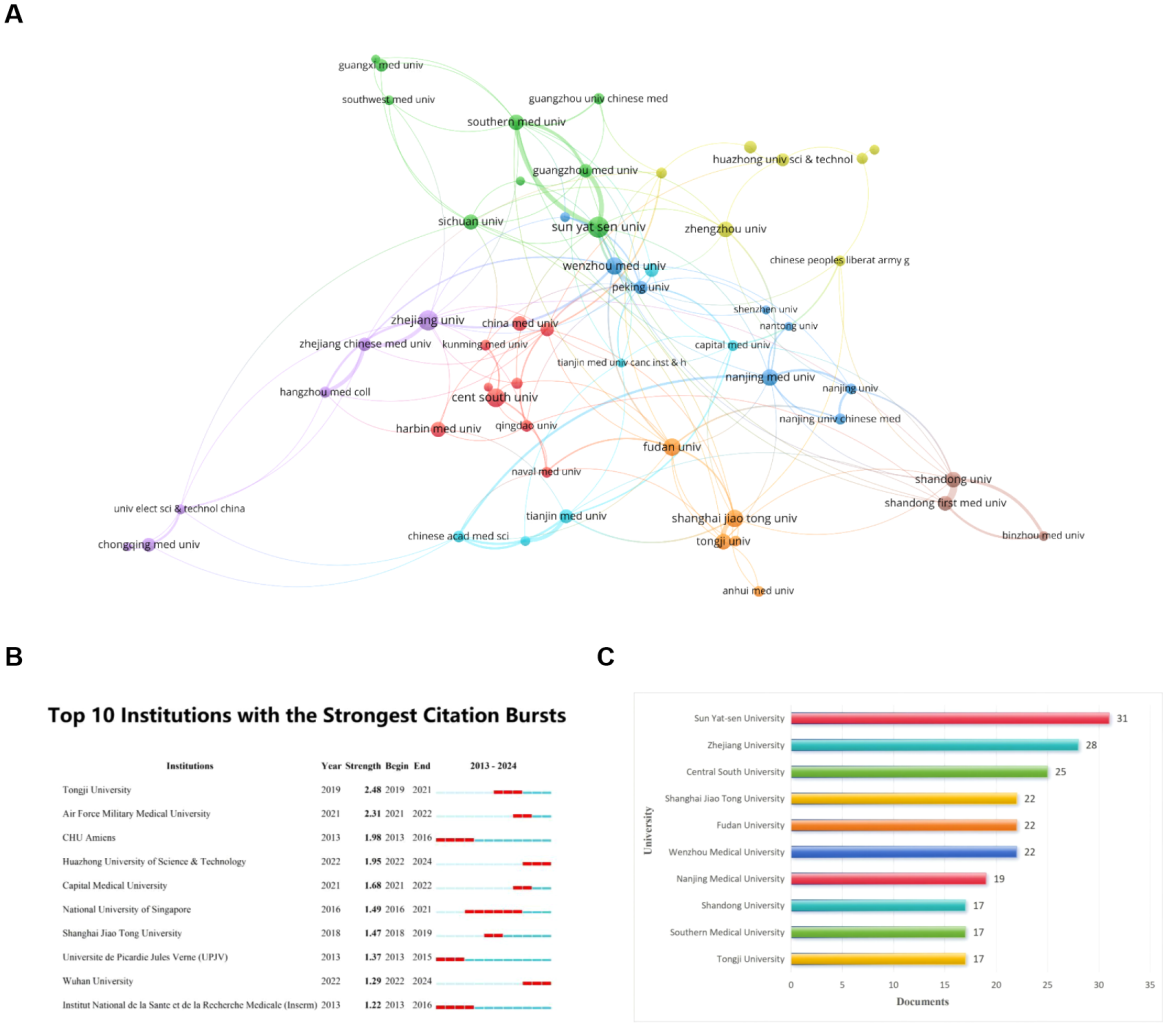
Visualization of institutions that performed research on ferroptosis in HCC. **(A)** Map of institutional Cooperation Contributions. The circles correspond to the number of publications from each institution, and the lines represent the collaborations between institutions. **(B)** Top 10 institutions with the most publication. **(C)** Top 10 institutions with the Strongest Citation bursts. From: VOSViewer.

There are 729 institutions that participated in the publication of papers related to ferroptosis in hepatocellular carcinoma, according to our data analysis of the collected literature. We selected 53 institutions with more than five publications for analysis by using VOSviewer. **Figure 4A** shows an expansive institution map with 53 nodes and 152 connecting links. The size of the node is positively correlated with the number of articles published in the institution, and these links reveal the cooperation relationship between institutions (The stronger the partnership, the wider the link). The cooperation between the agencies is relatively close, especially Sun Yat-sen University, Southern Medical University, and Guangzhou Medical University. The top ten contributing institutions, as outlined in **Figure 4B**, are Sun Yat-sen University, Zhejiang University, Central South University, Shanghai Jiao Tong University, Fudan University, Wenzhou Medical University, Nanjing Medical University, Shandong University, Southern Medical University, and Tongji University, they are originated from China.

The institutions with notable citation bursts were identified through CiteSpace (**Figure 4C**), and the highest-ranked university is Tongji University. Its citation bursts in 2019 and continued until 2021. Regrettably, this trend has not been consistently maintained over the last three years. Additionally, Huazhong University of Science & Technology and Wuhan University were identified as starting citation bursts in 2022 and continued until 2024. They still have a high level of enthusiasm.

### 3.4 Analysis of published journals and highly cited journals

By analyzing the journal publication data we collected, 261 journals have featured articles on ferroptosis in HCC. The table gives the top 10 journals considering the research number of this field (**Table 2**) and the top 10 journals with the highest number of citations (**Table 3**), ranked by frequency. We found Frontiers in Oncology (n=26, IF = 3.5) had the highest NP, followed by Frontiers in Pharmacology (n=20, IF = 4.4), Cell Death & Disease (n=15, IF = 8.1), Frontiers in Cell and Developmental Biology (n=14, IF = 4.6) and Frontiers in Immunology (n=12, IF = 5.7). These journals publish more articles on ferroptosis in HCC, and scholars can pay more attention. As can be seen from **Table 3**, Cell and Nature are the two most frequently cited journals, both with more than 1000 citations. It shows that these two journals are the core and authoritative journals in the field.

**Table 2:** Top 10 journals with the most publication.

Ranking	Journal	Documents	IF	H-index	JCR	Country
1	Frontiers in Oncology	26	3.5	83	2	Switzerland
2	Frontiers in pharmacology	20	4.4	86	2	Switzerland
3	Cell death & Disease	15	8.1	111	1	United Kingdom
4	Frontiers in cell and developmental Biology	14	4.6	53	2	Switzerland
5	Frontiers in Immunology	12	5.7	124	1	Switzerland
6	Redox Biology	12	10.7	88	1	Netherlands
7	Cell Death Discovery	12	6.1	28	1	United Kingdom
8	Biochemical and Biophysical Research Communications	12	2.5	263	1	United States
9	Biomedicine & Pharmacotherapy	10	6.9	92	1	France
10	Free Radical Biology and Medicine	10	7.1	265	1	United States

**Table 3:** Top 10 most cited journals.

Ranking	Journal	Citations	IF	H-index	JCR	Country
1	Cell	1206	45.5	776	1	United States
2	Nature	1075	50.5	1226	1	United Kingdom
3	Hepatology	860	12.9	361	1	United States
4	Cell death & Disease	774	8.1	111	1	United Kingdom
5	Cell Death and Differentiation	617	13.7	218	1	United Kingdom
6	Nature Communications	564	14.7	365	1	United Kingdom
7	Free Radical Biology and Medicine	522	10	265	1	United States
8	Cancer Research	511	12.5	449	1	United States
9	Biochemical and Biophysical Research Communications	495	2.5	263	1	United States
10	Cancer Letters	494	9.1	182	1	Netherlands

**Figure 5:**
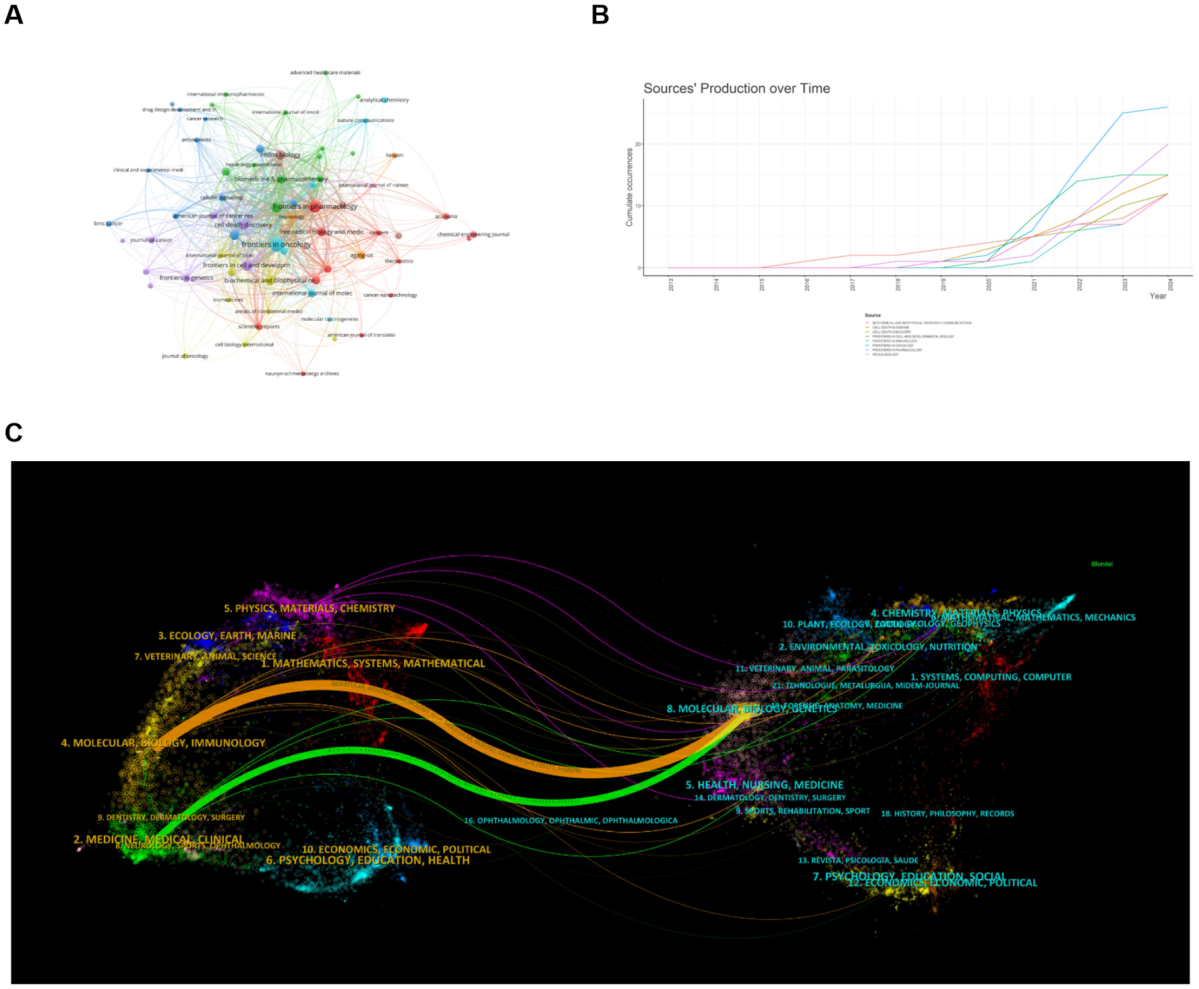
Analysis of published journals and highly cited journals on ferroptosis in HCC. **(A)** Map of journal collaborative collaborations. Circles represent journals, and lines represent interactions between journals. **(B)** Annual Publication Trends of Journals. **(C)** Dual-Map Overlay of Journals. The two main citation pathways in the figure reveal the information flow between citing journals and cited journals. From: VOSViewer, bibliometrix.

We set the minimum number of journals to 3 articles and selected 67 high-yield journals for VOSviewer analysis to generate a map of journal collaborative contributions (**Figure 5A**). As you can see from the graph, there are close partnerships between journals, with Frontiers in Pharmacology being the most active, which collaborated with 53 high-yield institutions. According to the journal’s annual publication growth chart (**Figure 5B**), Frontiers in Oncology has the fastest growth rate, with the highest number of publications in 2024, followed by Frontiers in Pharmacology. According to the journal’s double map overlay, the two main citation paths connecting the two maps reveal the flow of information between the journals (**Figure 5C**). On the left is the journal in which the paper is published, and on the right is the journal where the corresponding cited article is located. Articles published in Molecular, Biology, Immunology, Medicine, Medical, and Clinical all cite the literature on Molecular, Biology, and Genetics on the right.

### 3.5 Author and co-cited authors analysis

**Figure 6:**
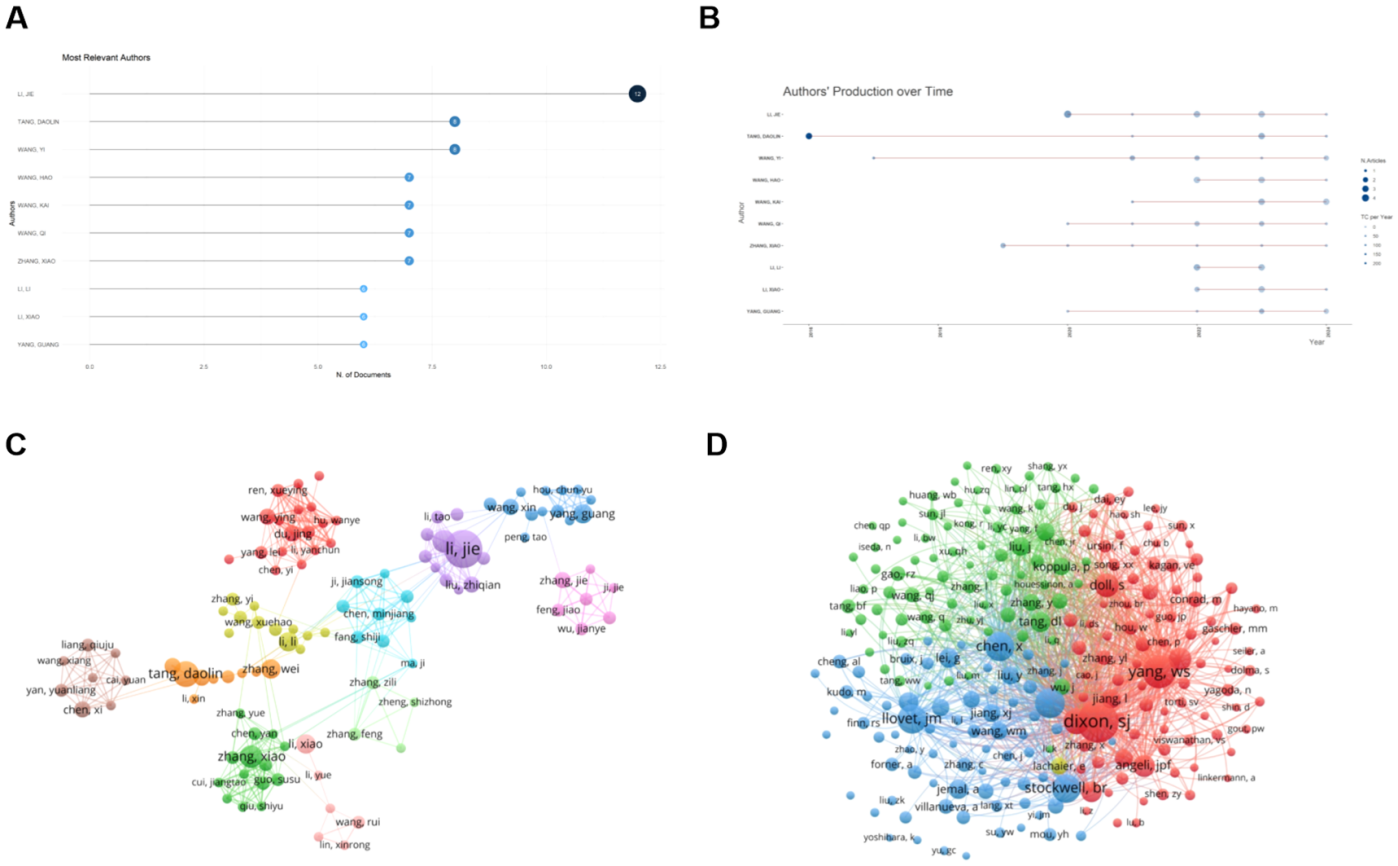
Analysis of authors and co-cited authors in ferroptosis research in HCC. **(A)** Top 20 productive authors. **(B)** Annual publication trends of the top 10 authors in publication terms. **(C)** The visualization map of author cooperation network. **(D)** Collaboration of co-cited authors. Circles represent the number of publications by authors, and lines represent interactions between authors. From: VOSViewer, bibliometrix.

A total of 4545 authors worldwide participated in these publications related to ferroptosis and HCC. Among them, Li Jie, who is from China, has the most published documents, with a total of 12. Followed by tang dao lin (n = 8) and wang yi (n = 8) (**Figure 6A**), and their annual publication variations are shown in **Figure 6B**. Tang Daolin was the first author to study the association between HCC and ferroptosis and continues to do so today. It is worth noting that Li Jie is the only author who has published more than ten documents, indicating that ferroptosis has less research on the pathogenesis of HCC and has excellent research potential. We set the minimum number of documents of an author to three, and 178 authors met the threshold. Using VOSviewer to generate a graph of author collaborative contributions (**Figure 6C**), nodes of the same color represent the same group of authors. We found that there was a close and strong working relationship between the same group of authors, while there was a less large-scale collaboration between different groups. **Figure 6D** shows that the top three authors with the most co-citations are Tang Daolin (n=363), Kang Rui (n=338), and Chen De (n=295). This shows that they have achieved convincing results and are authoritative.

### 3.6 Co-cited references analysis

**Figure 7:**
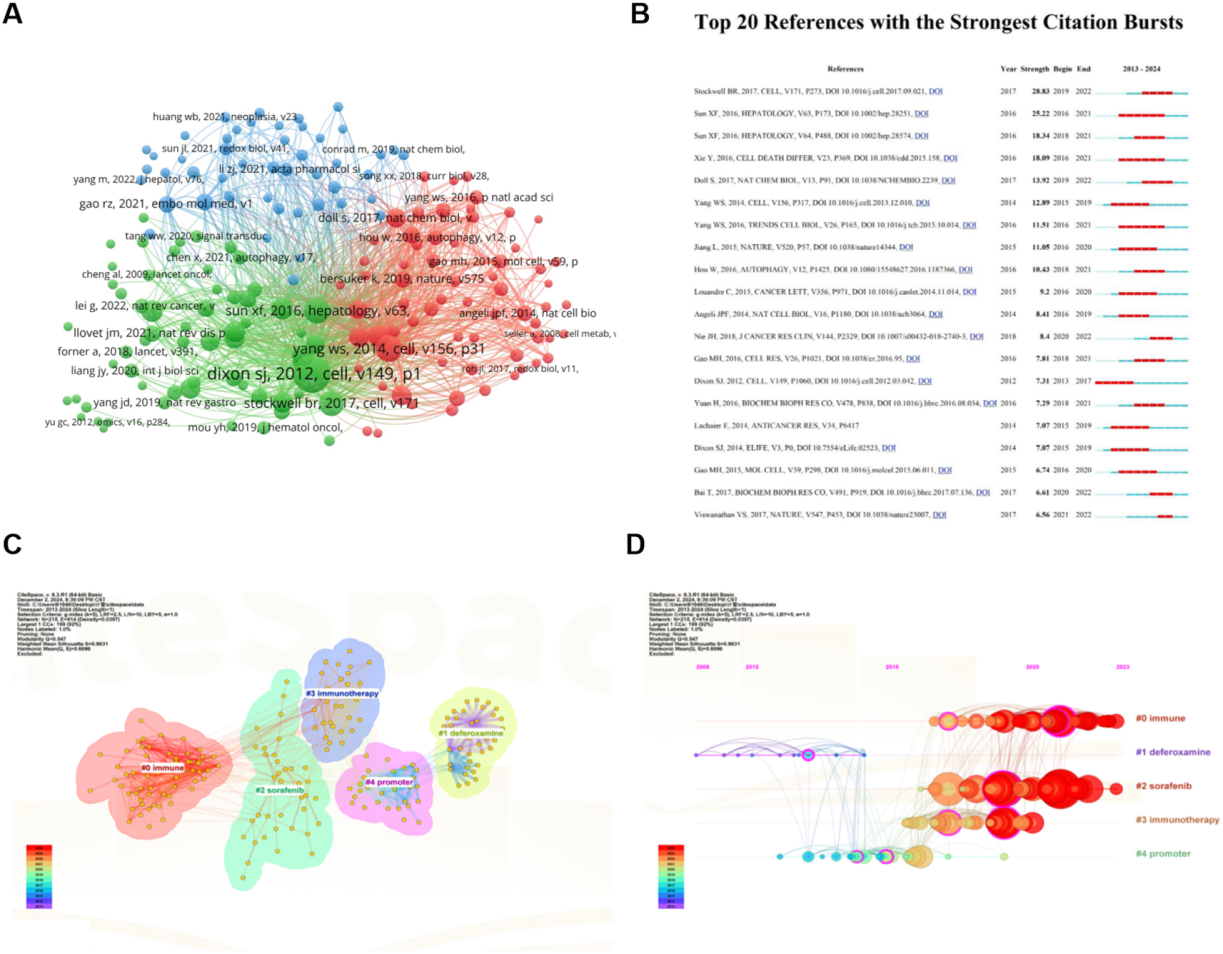
Analysis of Co-Cited References on ferroptosis research in HCC. **(A)** Network of 32 papers with co-citation frequency not less than 20. Circles are co-cited literatures. **(B)** The top 15 references with the strongest citation bursts. **(C)** Clustered network map of co-cited references. **(D)** The timeline view of co-cited references with their cluster labels on the right. From: VOSViewer, Citespace.

Among a total of 645 articles related to ferroptosis and HCC, there are 24,201 co-cited references. We selected 175 documents that were co-cited more than 20 times to generate a co-citation network diagram, which refers to a network of references with at least one paper that has been co-cited at the same time (**Figure 7A**). The top 10 references with the greatest number of co-citations are in **Table 4**, of which three are from China, 4 are from the United States, 2 are from France, and the remaining 1 is from Belgium. American scholars have made outstanding contributions to and influenced the field of ferroptosis in HCC. The most cited reference is “Ferroptosis: an iron-dependent form of nonapoptotic cell death” published by Scott J Dixon et al. In Cell in 2012, the concept of ferroptosis was first proposed. This is followed by “Regulation of ferroptotic cancer cell death by GPX4” published by Wan Seok Yang et al. in Cell in 2014. These two research articles have laid a certain foundation for research in the field of ferroptosis in HCC.

**Table 4:** The top 10 most co-cited references.

Rank	Title	Published year	First author	Journal	Co-citations	Publication types	Countries
1	Ferroptosis: an iron-dependent form of nonapoptotic cell death	2012	Scott J Dixon	Cell	354	Research	United States
2	Regulation of ferroptotic cancer cell death by GPX4	2014	Wan Seok Yang	Cell	178	Research	United States
3	Activation of the p62-Keap1-NRF2 pathway protects against ferroptosis in hepatocellular carcinoma cells	2016	Xiaofang Sun	Hepatology	169	Research	China
4	Ferroptosis: A Regulated Cell Death Nexus Linking Metabolism, Redox Biology, and Disease	2017	Brent R Stockwell	Cell	160	Review	United States
5	Metallothionein-1G facilitates sorafenib resistance through inhibition of ferroptosis	2016	Xiaofang Sun	Hepatology	126	Review	China
6	Iron-dependent cell death of hepatocellular carcinoma cells exposed to sorafenib	2013	Christophe Louandre	Int J Cancer	124	Research	France
7	Global Cancer Statistics 2020: GLOBOCAN Estimates of Incidence and Mortality Worldwide for 36 Cancers in 185 Countries	2021	Hyuna Sung	CA Cancer J Clin	123	Review	France
8	Pharmacological inhibition of cystine-glutamate exchange induces endoplasmic reticulum stress and ferroptosis	2014	Scott J Dixon	Elife	122	Research	United States
9	Broadening horizons: the role of ferroptosis in cancer	2021	Xin Chen	Nat Rev Clin Oncol	117	Review	China
10	Targeting Ferroptosis to Iron Out Cancer	2019	Behrouz Hassannia	Cancer Cell	111	Review	Belgium

A burst of citations is a sharp increase in the frequency of citations of an article over a specific period of time, which helps to unearth the latest research topics in related fields. **Figure 7B** shows the top 20 most cited references, with the blue line indicating the time interval at which the citation occurred and the red line indicating the period of time during which the highest citation was detected. Among them, the strength of four documents were more than 15 (4/20, 20%). For example, Stockwell BR et al. published the document entitled “Ferroptosis: A Regulated Cell Death Nexus Linking Metabolism, Redox Biology, and Disease” (21) on Cell ranking first with the largest strength (Strength = 28.83), followed by Sun XF et al. (Strength = 25.22) (22) in Hepatology, with only two strength over 20. It is worth noting that the strength of Sun XF’s two articles is greater than 15, ranking second and third, indicating that Sun XF has a significant influence in this field.

Cluster analysis of co-cited references has proven to be an effective way to discover hot topics in the field (23). The modular Q (0.547) is greater than 0.3, indicating that the clustering mechanism is significant, and Mean Silhouette (0.8631) values are greater than 0.7, indicating that the clustering results have high reliability. We performed a keyword-based cluster analysis of the cited literature (**Figure 7C**), and the leading cluster labels were immune, deferoxamine, sorafenib, immunotherapy, and promoter. The reference timeline view (**Figure 7D**) can clearly show the trend of research hotspots over time. As shown in the figure, the research on the initiation factors of cluster #1 (deferoxamine) and cluster #4 (promoter) started earlier, and there have been few studies in recent years, indicating that the research in this field is close to maturity. Cluster #0 (immune), Cluster #2 (sorafenib), and Cluster #3 (immunotherapy) contain a large number of red nodes, indicating that they are currently a hot topic of research in this field, that is to say, the field has shifted from the traditional mechanism to the treatment of HCC.

### 3.7 Keyword analysis

#### 3.7.1 Analysis of keyword co-occurrence

**Figure 8:**
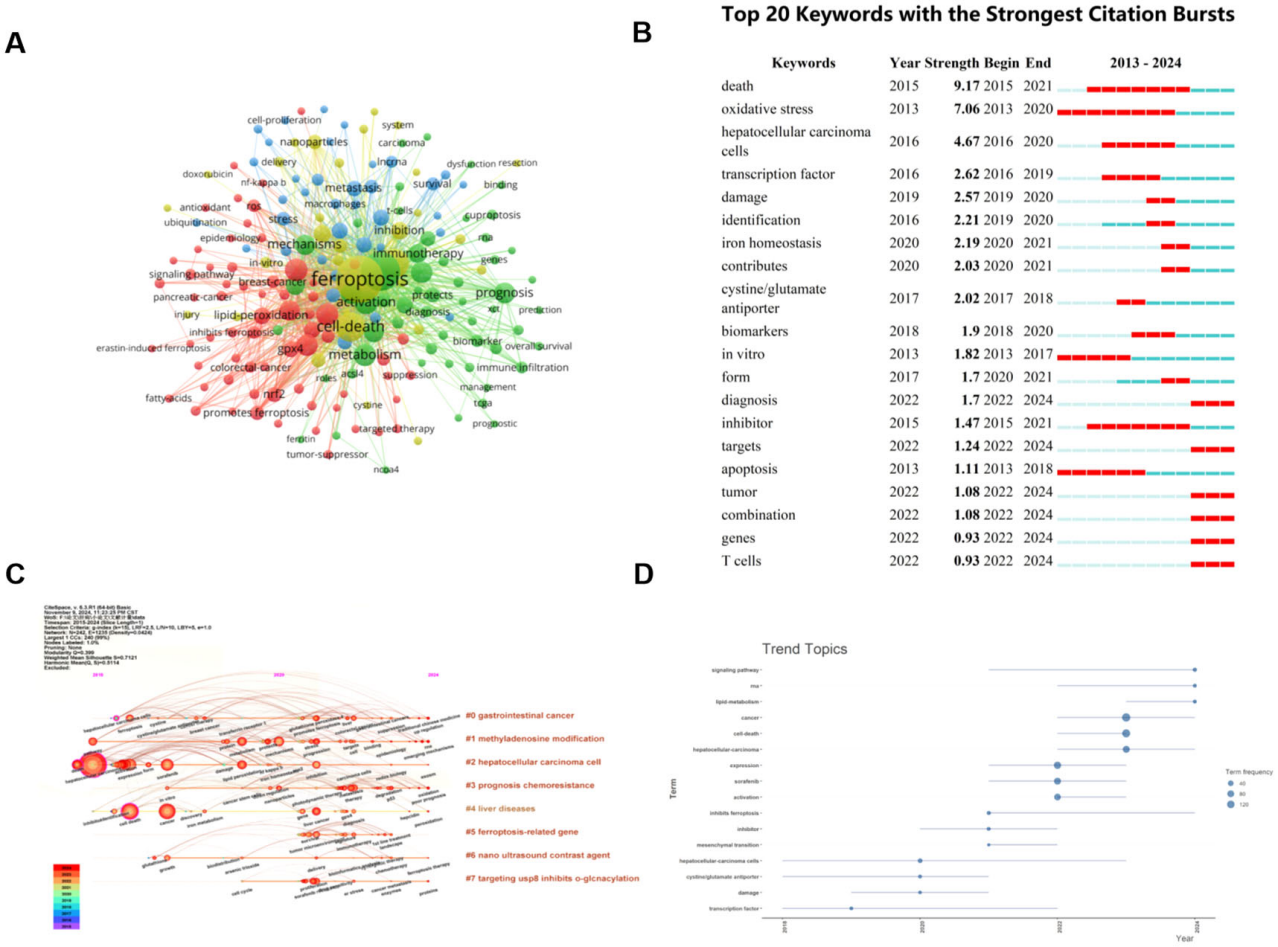
The keyword mapping of ferroptosis research in HCC. **(A)** A network map of keywords. Circles represent the frequency of keyword occurrences. **(B)** The top 20 keywords with the strongest citation bursts. **(C)** The timeline view of author’s keywords. **(D)** Trend topics based on keywords plus over time. From: VOSViewer, bibliometrix, Citespace.

Keywords are the central idea of the paper, and through the co-occurrence diagram of keywords, we can observe the research hotspots and directions in this field. We used VOSviewer to analyze the keywords with a minimum number of occurrences greater than 5 times among 2071 keywords to generate keyword co-occurrence graphs (**Figure 8A**); among them, the keywords with low relevance, such as death and cells, are removed. The top 10 high-frequency keywords are ferroptosis, hepatocellular carcinoma, cancer, cell death, sorafenib, mechanism, activation, apoptosis, metabolism, and iron. In a keyword co-occurrence network, four colors represent four different clusters, and the size of each node reflects the frequency of the keyword. Yellow clusters include ferroptosis, cell death, sorafenib, glutathione, therapy, and so on. The green cluster is mainly related to the pathogenesis, treatment, and prognosis of HCC, including hepatocellular carcinoma, mechanism, metabolism, activation, pathway, prognosis, immunotherapy, etc. The red cluster is related to the specific processes that include apoptosis, oxidative stress, lipid-peroxidation, ROS, and nrf2. Blue clusters include protein, metastasis, fibrosis, inflammation, and macrophage.

A timeline diagram can clearly show the relationship between keywords and their evolution, and at the same time, you can also observe the relationship with the timeline. CiteSpace was used to further analyze the keywords in the relevant literature, as shown in **Figure 8C**, eight tags were generated by keyword clustering, namely: cluster #0 “gastrointestinal cancer”, cluster #1 “methyladenosine modification”, cluster #2 “hepatocellular carcinoma cell”, cluster #3 “prognosis chemoresistance”, cluster #4 “liver diseases”, cluster #5 “ferroptosis-related gene”, cluster #6 “nano ultrasound contrast agent”, and cluster #7 “targeting usp8 inhibits o-glcnacylation”, they represent the current research hotspots and cutting-edge trends.

#### 3.7.2 Analysis of keywords citation burst

By examining the distribution of terms with the strongest citation explosion, it can predict the hot spots and future development dynamics in the field. Combined with the CiteSpace tool, we identified the top 20 keywords with the strongest citation bursts in the field over the past 10 years, as shown in **Figure 8B**. The intensity of the explosion of “death” is the most significant, with an index of 9.17, followed by “oxidative stress” and “hepatocellular carcinoma cells”. “Death” and “inhibitor” are the longest-lasting outbreaks, which first appeared in 2015 and showed rapid growth between 2015 and 2021. The keywords that have become popular in recent years are: “iron homeostasis”, “contributes”, “diagnosis”, “targets”, “tumor”, “combination”, “genes”, and “T cells”. And the outbreak of “diagnosis”, “targets”, “tumor”, “combination”, “genes”, and “T cells” has continued until now, which is a hot topic in this field. The keywords plus trend chart using R-bibliometric also reveals that the research hotspots have shifted to the following directions in recent years: signaling pathway, RNA, and lipid-metabolism (**Figure 8D**).

## 4 Discussion

### 4.1 General information

According to the search query we developed, a total of 938 articles were retrieved from the Web of Science between 1 January 2012 and 30 October 2024, and 645 articles were finally included through further screening, including 540 articles and 105 reviews. The articles were published in 261 journals by 4545 authors from 729 institutions in 32 countries, citing 24201 co-cited references from 2485 journals. The number of publications related to the ferroptosis of HCC has been increasing annually, especially in the past three years. A total of 518 publications have been published, accounting for 80.31% (**Figure 2**). Currently, China, the United States, and Japan are leading in this research field (**Table 1**). Specifically, China is the most published country, with the most citations and the top 10 research institutions and scholars in terms of publications are all from China (**Figures 4B**, **6A**). Among them, Sun Yat-sen University’s contribution is particularly outstanding, and the most published author is Li Jie, the only author with more than 10 publications. The United States is second, but it is worth noting that the 43 articles published in the United States have been cited 6331 times, and the average citation rate is the highest, as high as 147.23 (**Table 1**). This may have something to do with the fact that it has the most cited journal, Cell (**Table 3**).

### 4.2 Hotspot directions

**Figure 9:**
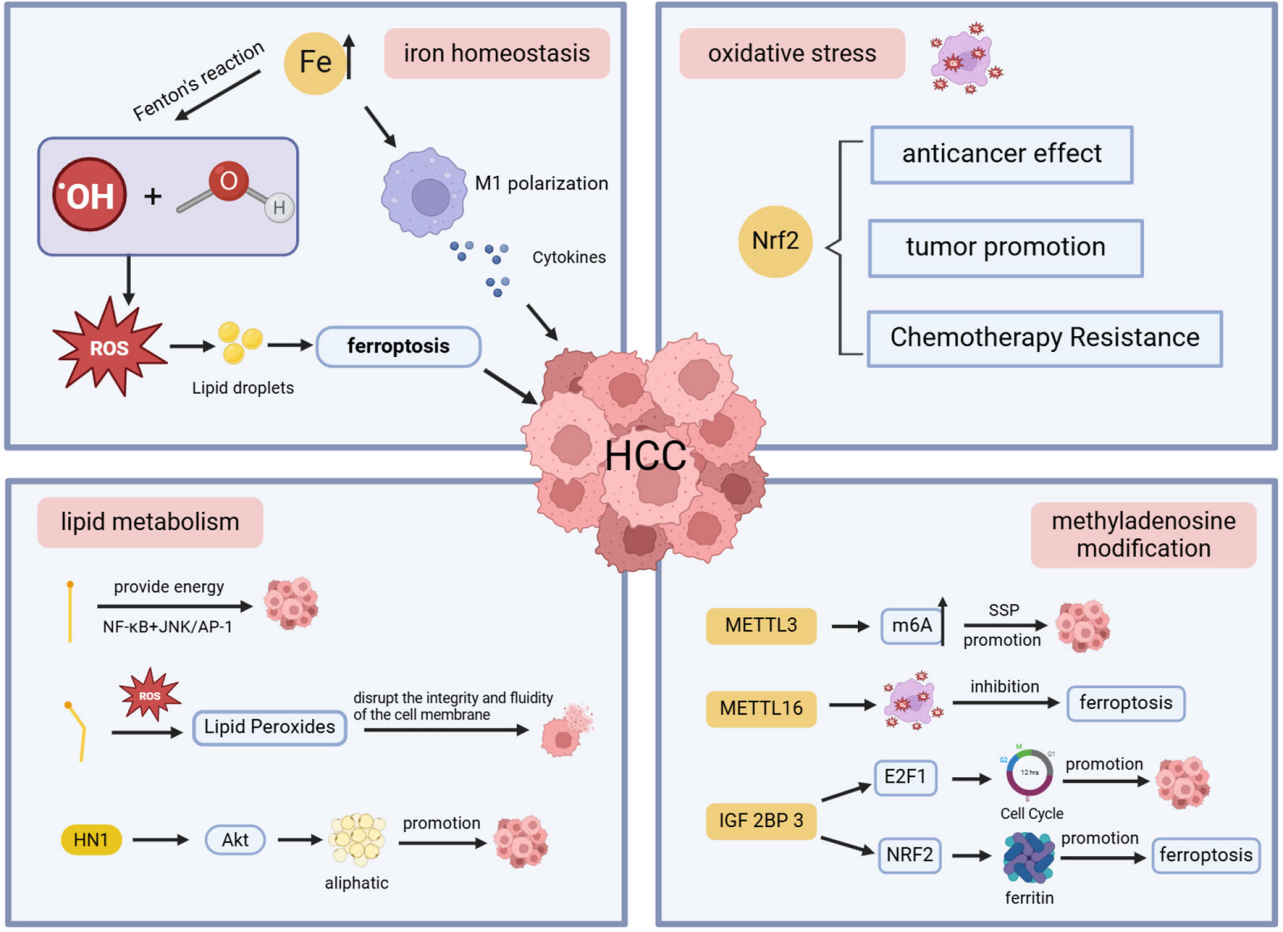
Schematic diagram of the mechanism of ferroptosis in HCC.

Based on the results of keyword co-occurrence and emergence analysis, we summarized the relationships between iron homeostasis, oxidative stress, lipid metabolism, methyladenosine modification, and ferroptosis (**Figure 9**), as well as current research hotspots and emerging trends in this field, including sorafenib, photodynamic therapy, and nano-contrast agents for ultrasound imaging. These findings may facilitate the identification of novel therapeutic targets for ferroptosis.

#### 4.2.1 Iron homeostasis

Iron is an indispensable trace element in the human body, which participates in cell division, metabolism, and other physiological activities (22, 23). The liver, as the main place of iron storage, can maintain iron homeostasis by producing and secreting the main regulator of hepcidin (24). Iron levels in the body are strictly controlled to avoid iron overload and iron-related toxicity (25), and an imbalance of iron homeostasis can lead to the occurrence of a variety of diseases. The excess free iron in the liver produces hydroxides and hydroxyl radicals through the Fenton reaction, which exceeds cellular defenses and leads to the development of liver cancer (26). When there is an imbalance between the production and scavenging of ROS, it leads to lipid peroxidation on the cell membrane, triggering ferroptosis (27). There is a wonderful relationship between ferroptosis and HCC: iron accumulation promotes the development of HCC, and even high levels of iron in the diet can increase the risk of HCC (28), while activation of ferroptosis hinders HCC cell proliferation (29). This suggests that tumor cells increase iron levels during rapid proliferation to meet their metabolic needs (30). At the same time, they try to block oxidative stress by activating peroxidase 4 (GPX4), SLC11A2, etc., which are involved in ferroptosis regulation, thereby preventing cell death and promoting the progression of HCC (31, 32). Iron chelator can not only reduce the content of extract, but also remove ROS and play an antioxidant role, thereby delaying the progression of HCC (33).

Iron metabolism in the tumor microenvironment is intricately connected to the immune system and is involved in the occurrence, development, and prognosis of HCC by influencing macrophage polarization (34, 35). Iron excess promotes M1 polarization of macrophages, secretes various cytokines, and destroys HCC cells, while iron deficiency usually leads to M2 polarization, which can promote HCC cell growth (36, 37). In the liver, iron accumulation promotes oxidative stress, leading to increased accumulation of lipid droplets. The disruption of iron homeostasis also affects lipid drop formation and size, leading to liver cell damage (38, 39). Therefore, dynamic monitoring of ferrous ion levels and lipid droplet changes is essential to assess disease severity and progression. NRFep stands out for its excellent stability in cell and animal models, opening up new ideas for diagnosing and treating diseases (40).

#### 4.2.2 Oxidative stress

Oxidative stress is damage to cells and tissues caused by the production of ROS in living organisms that exceed their scavenging effect (41). The body’s antioxidant system can remove excess ROS, maintain redox balance, and prevent cell death caused by oxidative stress (42). Oxidative stress can disrupt normal cell function and interfere with signaling pathways, which is a pivotal factor in the development of liver cancer of various etiologies (43). Nuclear factor erythroid 2-related factor 2 (Nrf2) is a transcription factor with cytoprotective properties that protects against oxidative damage triggered by damage and inflammation by regulating the expression of antioxidant proteins (44). Nrf2 has a dual role in HCC. Nrf2 has anticancer effects, which can attenuate the effects of oxidative stress and reduce the risk of mutations and cancer. When Nrf2 accumulates abnormally in the nucleus, it not only prevents oxidative stress but also promotes the survival and growth of HCC cells, leading to the progression and aggressiveness of the disease (45). Chemoresistance in HCC is also closely related to Nrf2 (46). ROS in cells is mainly produced by mitochondria, and FAN has been shown to have an anti-tumor effect, which can increase the amount of ROS production and induce oxidative stress by impairing mitochondrial function in HCC cells, thereby promoting apoptosis and even death of tumor cells (47).

#### 4.2.3 Lipid metabolism

Lipid metabolism, including the synthesis, storage, and breakdown of lipids, maintains cellular homeostasis, and the liver is its main organ (48). Abnormal lipid metabolism may lead to excessive accumulation of lipids within cells, creating a lipotoxic environment (49, 50). Excessive accumulation of specific lipid peroxides can disrupt the integrity and fluidity of cell membranes, hinder signaling and energy supply, and directly induce ferroptosis (51). The accumulation of lipid peroxides plays a decisive role in ferroptosis. Liyuan Hao et al. determined that the expression of lipid metabolism-related genes is closely related to the immune microenvironment of HCC patients and plays an essential role in predicting the prognosis of HCC through a prognostic risk model (52). Apigenin can down-regulate the gene of lipid metabolism, induce cycle arrest and apoptosis of HCC cells, inhibit its growth, promote the necrosis of HCC cells, and delay the progression of HCC (53). Studies have found that the key metabolites involved in glycerol phospholipid metabolism in HCC patients, such as phosphatidylcholine and lysophosphatidylcholine, are significantly up-regulated, further emphasizing the importance of lipid metabolism in the progression of HCC and is expected to become an important indicator to improve the early diagnosis rate of HCC and improve the prognosis of patients (54). Hua Jin et al. found that the high expression of HN1 in HCC tissues can trigger the Akt signaling pathway and promote adipogenesis, which leads to the proliferation, migration, and invasion of HCC cells. Inhibition of the expression of HN1 can effectively inhibit the growth of HCC cells and is expected to be a new therapeutic strategy (55). Studies have shown that saturated fatty acids can promote the proliferation of HCC cells, while unsaturated fatty acids play an inhibitory role (56).

#### 4.2.4 Methyladenosine modification

Epigenetic modification is the main regulator of gene expression, and RNA modification can promote tumor growth, metastasis, and chemotherapy resistance (57, 58). N6-methyladenosine (m6A) modification is the most characteristic RNA modification, and its mediated serine synthesis pathway (SSP) plays a vital role in the development and treatment resistance of HCC. Studies have proved that the upregulation of m6A methyltransferase METTL 3 drives the progression of HCC (59), and the specific METTL 3 inhibitor STM 2457 can attenuate SSP, induce the occurrence of oxidative stress, effectively inhibit the growth of HCC cells, and improve the sensitivity of HCC cells to sorafenib (60). At present, most scholars in various countries have elaborated on the mechanism of ferroptosis from the perspectives of iron accumulation, oxidative stress, and lipid peroxidation, and few have studied it from a new perspective. METTL16 was recently identified as the second m6A writer, a novel ferroptosis repressor in HCC cells that can promote cell viability and tumor progression (61). Jialin Wang et al. discovered a novel METTL16-SENP3-LTF signaling axis that can modulate ferroptosis and participate in the progression of HCC, becoming a promising therapeutic strategy by targeting this axis to disrupt ferroptosis and ferroptosis susceptibility (62). Insulin-like growth factor 2 mRNA-binding protein 3 (IGF2BP3) is an important m6A reader with enhanced expression in liver patients and inhibits ferroptosis, promoting HCC cell malignant behavior and macrophage M2 polarization (63). Some studies have shown that IGF2BP3 may regulate the cell cycle by controlling the stability and transcription of the transcriptional activator E2F1 (64), promote the proliferation, colony formation, and invasion of HCC cells, and is expected to be a novel marker for predicting ferroptosis, HCC immune response and prognosis (65). Zhihua Lu et al. found that IGF2BP3 read m6A modification to promote the stability of NRF2 mRNA from *in vitro* and *in vivo* experiments, further revealing that the IGF2BP3-NRF2 axis can affect ferritin deposition during the pathogenesis of HCC, thereby regulating ferroptosis, which provides the possibility of improving the sensitivity of hepatocellular carcinoma cells to sorafenib (66).

#### 4.2.5 Sorafenib

Sorafenib is a multi-targeted tyrosinase kinase inhibitor for treating advanced HCC, prolonging median overall survival by 6.5 months by inhibiting tumor proliferation and angiogenesis (67, 68). Existing studies have confirmed that sorafenib selectively targets a cystine-glutamate antiporter (system Xc-), triggering ferroptosis and thereby effectively eliminating tumor cells (69). HCC cells exhibit a high sensitivity to ferroptosis due to the high demand for iron ions (70). Regulator X1 (RFX1) inhibits the activity of system Xc- by upregulating the expression of BECN1, induces ferroptosis in HCC cells, and enhances the anticancer activity of sorafenib (71). Sorafenib is the first-line treatment for advanced HCC, but its resistance remains a challenge. Yong Zhao et al. found that liver-specific lncRNA HNF4A-AS1 was down-regulated in sorafenib-resistant cells, reducing the accumulation of intracellular polyunsaturated fatty acids (PUFAs), thereby promoting resistance to sorafenib-induced ferroptosis, further suggesting that HNF4A-AS1 is expected to be a potential target for inhibiting sorafenib resistance (SR) (72). Studies have shown a strong relationship between ferroptosis obstruction and SR in HCC (73). Circ_0001944 targets the miR-1292-5p/FBLN2 axis to inhibit ferroptosis, leading to SR. Circ_0001944 up-regulation in sorafenib-resistant HCC cells is a putative target for reversing SR in HCC, which is expected to provide a new direction for sorafenib sensitization (74, 75). Xiangbo Huang et al. found that HCC cells can promote the secretion of IL4 by sorafenib and trigger the formation of MET, which promotes SR by inhibiting ferroptosis in HCC cells, suggesting that targeting the IL4/PADI4/MET axis in HCC can reduce or prevent SR (76). Shiwen Ma et al. found that Glutathione S-transferase α1 (GSTA1) was highly expressed in HCC drug resistance models, and GSTA1 could promote the entry of CTNNB1 in the cytoplasm into the nucleus and activate the transcriptional activity of GSTA1, promote the expression of GSTA1, and form a positive feedback loop. GSTA1 regulates SR by exerting its peroxidase function, and inhibition of GSTA1 expression can enhance the sensitivity of sorafenib, which is expected to become a new therapeutic target (77). DDX11 antisense RNA 1 acts as a resistance catalyst by activating the Nrf2-Keap1 pathway in HCC to prevent sorafenib-triggered ferroptosis (78). In recent years, more and more scholars have devoted themselves to studying the resistance mechanism of sorafenib and identified a number of related therapeutic targets, which provides a new perspective and direction for improving the sensitivity of sorafenib in the future.

#### 4.2.6 Photodynamic therapy

Photodynamic therapy (PDT) is a non-invasive treatment modality that stimulates photosensitizers (PS) at targeted sites by irradiating specific wavelengths of light to cause ROS accumulation and induce cell death (79). Compared with traditional chemotherapy regimens, PDT is effective in reducing side effects and preventing the development of drug resistance (80). Jianbing Du et al. designed an exosome with three parts, consisting of CD47, Erastin, and Rose Bengal. Drug-loaded exosomes strongly induced ferroptosis in tumor cells after 532 nm laser irradiation, and their application to the HCC xenograft model showed efficient anti-tumor effect and low toxicity and side effects (81). Chonggao Wang et al. reported a nano platform for the co-delivery of sorafenib, Ce6, and MnO2 for chemotherapy/PDT/PTT combination therapy, which inhibits the proliferation of HCC cells, improves anti-tumor efficacy and reduces drug resistance by alleviating tumor hypoxia, increasing thermogenesis and ROS production, promoting sorafenib tumor accumulation and distribution, and inducing ferroptosis (82). To overcome SR, Rui Sun et al. proposed a nano-platform with an “on/off” function, SR780@Fe-PAE-GP, which enhances the synergistic effect of death and photodynamic therapy with a tumor suppression rate of up to 98% (83). In recent years, with the deepening of research on photodynamic therapy, the combination of photodynamic therapy and traditional treatment in the treatment of HCC has advantages and has become more and more popular (84).

#### 4.2.7 Nano-ultrasound contrast agents

The advent of nano-ultrasound contrast agent (Nano-USCA) overcomes the shortcomings of traditional ultrasound only for intravascular imaging, which promotes imaging within tumor tissues and improves drug loading capacity so that drugs can accumulate at the tumor site to enhance the therapeutic effect. The combination of ultrasound and son dynamic therapy can deliver medicines in a targeted manner, improve imaging clarity, and monitor tumor changes in real time, which has great potential for future applications (85). Programmed cell death protein 1 (PD-1) is an immune checkpoint molecule that effectively reduces immune function and enhances tolerance (86). Yijie Qiu et al. reported a Nano-USCA (arsenic trioxide (ATO)/PFH NPs@Au‐cRGD) that achieves ultrasound imaging and stimulates an immune response by ferroptosis and combined chemotherapy-photothermal therapy. The combination of the ATO/PFH NPs@Au‐cRGD nano drug delivery system and anti-programmed cell death 1 (PD-L1) antibody can achieve synergistic treatment of HCC, which can effectively inhibit liver orthotopic tumors and lung metastases and can intuitively evaluate the treatment effect through visual imaging (87).

In recent years, scholars from all over the world have been enthusiastic about the research of nanomaterials, in order to achieve better results in the treatment of HCC. Due to the antioxidant function of the complex tumor microenvironment, the killing effect of ferroptosis on tumor cells is not significant (88). Kunzhao Huang et al. synthesized the GOx/EC@Fe3O4@CCM nanometer platform, which reduces the escape effect of ROS and maintains a higher level of intracellular oxidation, thereby inducing persistent and intense ferroptosis, showing the advantage of inhibiting and killing HCC cells (89). Rui-Rui Zhao et al. designed a metal-coordinated carrier-free nanodrug (USFe3+LA NPs) assembled by UA, SRF, and Fe to exert anti-tumor effects through a combination of ferroapoptosis, chemotherapy, and chemokinetic cocktail therapy. USFe3+LA NPs can improve the stability of nanodrugs, accumulate in tumor cells, and achieve the purpose of preventing tumor growth and metastasis without harming normal cells (90). The MIL-100@Apa@MPN drug delivery system created by Fengyi Yang et al. is used to induce ferroptosis, which has good safety, sustained release, and pH reactivity, which can better solve the shortcomings of insufficient targeting of apatinib and improve the therapeutic efficacy (91). Zhi-Yong Liu et al. constructed a stable nano platform (PE@PTGA) to solve the problem of poor water solubility of elastin. Self-assembled nanoparticles can enter HCC cells and release protoporphyrin IX (PpIX) and elastin. Under photostimulation, PpIX exerts hyperthermia and live ROS to induce apoptosis; Erastin acts as a potent ferroptosis trigger to induce ferroptosis in HCC cells, and the accumulated ROS can further promote ferroptosis and exert synergistic anti-tumor effects, thereby inhibiting the proliferation of HCC cells (92).

### 4.3 Research trends

Ferroptosis is an iron-dependent form of regulated cell death. Fe³^+^ enters the cell through transferrin receptor 1 (TFR1), is reduced to Fe²^+^ by STEAP3, and Fe²^+^ acts as a catalyst in the Fenton reaction, generating a large amount of ROS, leading to the formation of lipid peroxides. These lipid peroxides accumulate in the cell membrane, causing damage to its fluidity and integrity, ultimately triggering cell death. Concurrently, glutathione GPX4 uses glutathione (GSH) to reduce lipid peroxides to non-toxic lipid alcohols, which is a key antioxidant defense mechanism against ferroptosis. When the function of GPX4 is inhibited or GSH levels are reduced, cells become more susceptible to ferroptosis. Additionally, System Xc^-^ composed of SLC7A11 and SLC3A2, mediates cystine uptake, which is crucial for maintaining GSH levels. Its inhibition reduces GSH synthesis, increasing the cell’s vulnerability to ferroptosis.

As research into the mechanisms of ferroptosis in HCC continues to advance, an increasing number of molecular targets associated with ferroptosis have been identified. These targets modulate ferroptosis through various biological pathways, offering potential solutions to drug resistance and providing new perspectives and research directions for the clinical treatment of hepatocellular carcinoma. Thermal ablation techniques, such as microwave ablation and radiofrequency ablation, have been widely applied in the clinical treatment of HCC. However, there is a concern that incomplete ablation may lead to recurrence and metastasis. Jiayan Huang and colleagues found that after sub-lethal heat stress (HS), the glycolysis in HCC cells increases, and lactate production rises, which helps to promote the metastasis of HCC cells. Concurrently, the level of H3K18la modification increases, enhancing the transcriptional activity of NFS1 and reducing the cells’ susceptibility to ferroptosis, further promoting the metastasis of HCC cells. Additionally, the study discovered that knocking down NFS1 can inhibit the metastatic ability of HCC cells and increase their vulnerability to ferroptosis, thereby enhancing the therapeutic sensitivity to oxaliplatin. Therefore, the combined application of this therapeutic strategy has the potential to further improve clinical outcomes (93). Research has discovered that Scavenger Receptor Class A Member 5 (SCARA5) promotes the autophagic degradation of ferritin, increasing the concentration of biologically available ferrous ions within cells, thereby advancing the occurrence of ferroptosis. Conversely, the deficiency of SCARA5 reduces ferrous ion levels, inhibits ferroptosis, and leads to SR in HCC cells. Additionally, the study found that the expression levels of SCARA5 are positively correlated with the sensitivity of HCC patients to sorafenib. These findings offer new insights into the molecular mechanisms underlying SR in HCC cells (94). Lenvatinib is a multi-targeted tyrosine kinase inhibitor used for the treatment of advanced HCC, but its efficacy when used as a monotherapy is limited. Shiyu Zhang and colleagues found that Checkpoint kinase 1 (CHK1) is abnormally overexpressed in HCC and is associated with tumor progression and poor prognosis. Lenvatinib can increase the levels of phosphorylated CHK1 (p-CHK1) protein in HCC cells, and when used in combination with the CHK1 inhibitor Prexasertib, it exhibits synergistic anti-tumor effects in both HCC cells and *in vivo* experiments. This combined therapy primarily functions by upregulating the expression levels of ALOX15, inducing ferroptosis. This discovery provides a new combined treatment strategy for HCC (95). SLC7A11 and GPX4 are negative regulators of ferroptosis. Compared to normal liver tissue, both are upregulated in HCC and exhibit lower levels of ferroptosis. Research has found that atorvastatin can induce ferroptosis in HCC cells by increasing the levels of ROS and decreasing the expression of genes that negatively regulate ferroptosis, thereby inhibiting cancer cell growth and migration (96).

Shiqi Liu and colleagues found that neuroepithelial-like protein 2 (NELL2), by regulating the Notch signaling pathway, inhibits epithelial-mesenchymal transition (EMT) and promotes ferroptosis, significantly suppressing the proliferation, migration, and invasion of HCC cells. The study suggests that NELL2 may serve as a novel biomarker and potential therapeutic target for HCC (97). Analysis from multiple databases indicates that the EGF domain-specific O-linked β-N-acetylglucosamine transferase (EOGT) is significantly upregulated in HCC tissue and is associated with poor patient prognosis and drug resistance. HEY1 is a key factor in the regulation of ferroptosis and proliferation in HCC cells by EOGT. Experimental research by Zhe Yu and colleagues found that EOGT affects the expression levels of HEY1 through the Notch signaling pathway, and HEY1, acting as a transcriptional activator, can regulate the expression of SLC7A11. Reducing the expression of HEY1 exacerbates the increase in ferroptosis-related indicators caused by EOGT knockdown, further inhibiting the proliferation of HCC cells; conversely, overexpressing HEY1 can partially reverse these changes. This study is the first to reveal the EOGT-HEY1-SLC7A11 regulatory axis, clarifying the role of EOGT in inhibiting ferroptosis and promoting tumor proliferation in HCC, providing new potential therapeutic targets and strategies for the treatment of HCC (98). Han Gao’s team discovered through database analysis that HSPA4 is significantly upregulated in HCC tissue and can serve as an effective biomarker for early screening of HCC. The research found that the LINC01004/hsa-miR-125b-2-3p/HSPA4 axis inhibits ferroptosis in HCC cells by affecting GPX4 activity and iron metabolism, thereby influencing tumor progression. This axis is associated with the clinical staging of HCC, offering new targets for HCC treatment (99). Recent research has found that YBX1 inhibits ferroptosis and promotes the progression of HCC through the RNF115-DHODH signaling axis. RNF115, acting as an E3 ubiquitin ligase, promotes the ubiquitination of dihydroorotate dehydrogenase (DHODH) at Lys27 (K27) and inhibits its autophagic degradation, thereby resisting ferroptosis. Furthermore, NOP2/Sun RNA methyltransferase 2 (NSUN2) enhances the circularization and translation efficiency of RNF115 mRNA by modifying its m5C. This discovery provides new insights into the regulatory mechanisms of ferroptosis in HCC (100). Jinni Yao and colleagues discovered that GINS4 is highly expressed in HCC. Silencing GINS4 can inhibit the proliferation and cell cycle of liver cancer cells. Furthermore, GINS4 promotes ferroptosis in HCC cells by regulating the PI3K/AKT signaling pathway through binding with the POLE2 protein. *In vivo* experiments indicate that the absence of GINS4 can suppress tumor growth in HCC, increase the expression of GPX4, and decrease the levels of Ki67, while also reducing the activity of the POLE2/PI3K/AKT signaling pathway (101). Wei Peng and colleagues discovered that the expression levels of AIFM2 and IGF2BP1 are significantly elevated in HCC tissue and are associated with poor patient survival rates, with a positive correlation observed between IGF2BP1 and AIFM2. IGF2BP1 stabilizes AIFM2 mRNA through m6A modification, increasing its expression, which in turn promotes glycolysis in HCC cells and inhibits ferroptosis. Concurrently, *in vivo* experiments have shown that knocking down IGF2BP1 or AIFM2 can effectively suppress tumor growth and metastasis. This study reveals the significance of the IGF2BP1/AIFM2 axis in the progression of HCC (102). Liuzheng Li and colleagues, using a combination of bioinformatics analysis and *in vitro* experiments, discovered that N-myc downstream-regulated gene 1 (NDRG1) is highly expressed in HCC and significantly associated with poor patient prognosis. Furthermore, NDRG1 can mitigate Erastin-induced ferroptosis and promote tumor progression, with eight genes, including HSPA8 and CDH1, potentially being part of its underlying molecular mechanisms. However, the study has limitations, including the lack of *in vivo* experiments (103).

### 4.4 Chinese medicine molecules in the ferroptosis of HCC

Sorafenib, a first-line treatment drug for advanced HCC, can exert antitumor effects by promoting ferroptosis. However, its clinical application is often limited by severe adverse reactions and drug resistance. With the emergence of the concept of ferroptosis, research on treating HCC through ferroptosis regulation using Traditional Chinese Medicine has gradually increased. A large number of studies have shown that Chinese medicine molecules can not only inhibit the proliferation, migration, and metastasis of HCC cells but also suppress tumor growth, with fewer side effects. This article briefly summarizes the specific molecular mechanisms of Chinese Medicine molecules in regulating ferroptosis to treat HCC, providing a safer and more effective treatment option for patients with advanced liver cancer. (**Table 5**).

**Table 5:** Chinese medicine molecules promoting ferroptosis for HCC.

Chinese medicine molecules	Source	Structure	Molecular Formula	Description	*In vivo*/ *In vitro*	Model	Related targets	References
Artesunate	*Artemisia annua* L.	C_19_H_28_O_8_	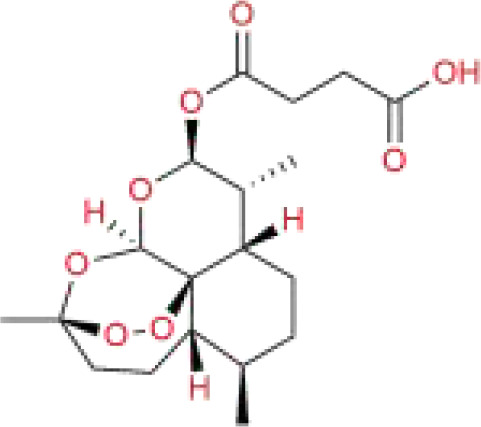	sesquiterpene lactones	*In vitro*/*In vivo*	HepG2 cells, Huh7 cells, HL-7702 cells. The HepG2 cells were subcutaneously injected into the axillary region of BALB/c nude mice.	–	(105)
Solamargine	*Solanum nigrum* Tausch ex Dunal	C_45_H_73_NO_15_	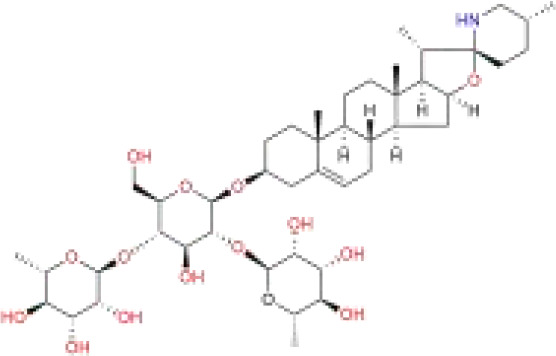	glycoalkaloid	*In vitro*/*In vivo*	HepG2 cells, Huh7 cells. The Huh-7 cells were subcutaneously injected into the right flank of BALB/c nude mice.	STAT 1/MTCH 1	(106)
Chlorogenic acid	*Eucommia ulmoides* Oliv., *Lonicera japonica* Thunb.	C_16_H_18_O_9_	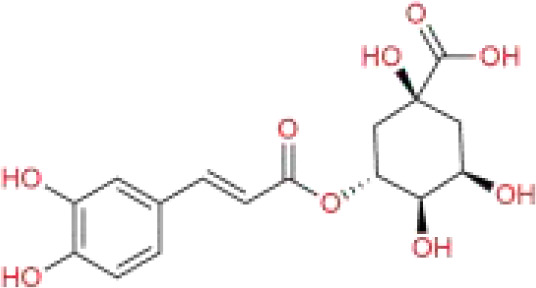	polyphenols	*In vitro*	HT-29 cells.	PTGS 2/AKR 1C 3/GPX 4	(108)
Juglone	Juglandaceae plants	C_10_H_6_O_3_	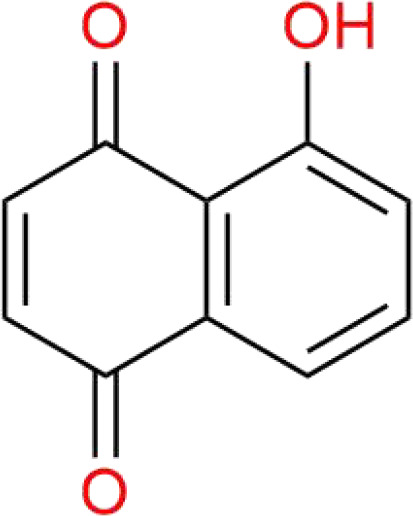	naphthoquinone	*In vitro*/*In vivo*	Huh7 cells, PLC cells. The PLC or Huh7 cells were subcutaneously injected into the right flank of BALB/c nude mice.	FOSL1-HMOX1	(109)
Faberidi- lactone A	*Inula japonica* Thunb.	C_15_ H_20_O_5_	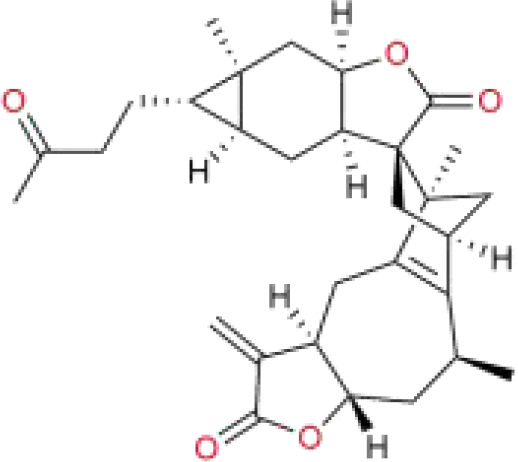	sesquiterpenoid dimer	*In vitro*/*In vivo*	HepG2 cells. HepG2 cells were microinjected into the yolk sacs of zebrafish embryos.	GSH	(110)
Polyphyllin I	*Paris polyphylla* Sm.	C_44_H_70_O_16_	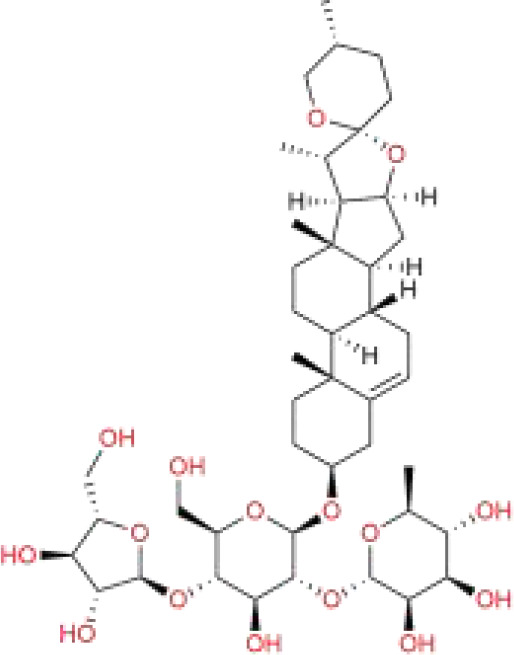	saponins	*In vivo*	The MHCC97H cells were subcutaneously injected into the right axilla of BALB/c nude mice.	Nrf 2/HO-1/GPX 4	(111)
Iberverin	*Brassica oleracea* var. *Capitata* L.	C_5_H_9_NS_2_	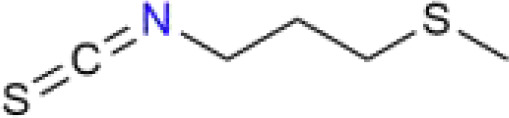	alkaloids	*In vitro*	HLE cells, HCCLM3 cells.	SLC7A11, GPX4	(113)
Auraptene	citrus plants and fruits	C_13_H_12_O_3_	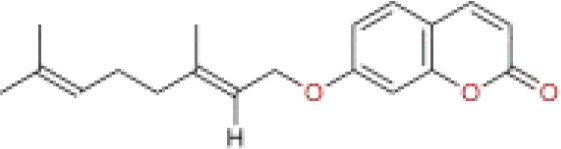	coumarin	*In vitro*	HCCLM3 cells, HLE cells.	SLC7A11	(114)
Ginsenoside RK1	*Ginseng* Alph.Wood	C_42_H_70_O_12_	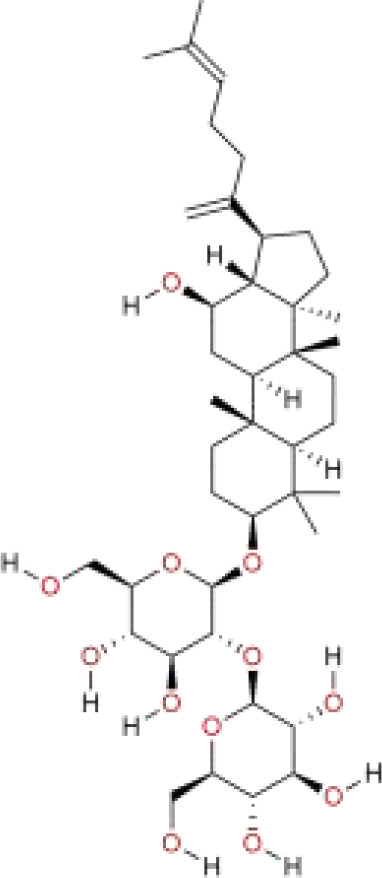		*In vitro*	HepG2 cells, Hep3B cells.	FSP1	(115)
curcumin	*Curcuma longa* L.	C_21_H_20_O_6_	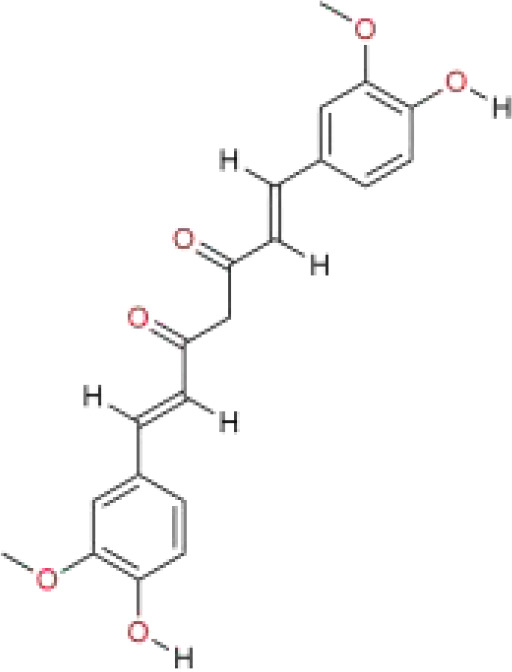	polyphenols	*In vitro*	HepG2 cells, SMMC7721 cells.	ACSL4	(116)
Cyclovirobuxine D	*Buxus microphylla* Siebold & Zucc.	C_26_H_46_N_2_O	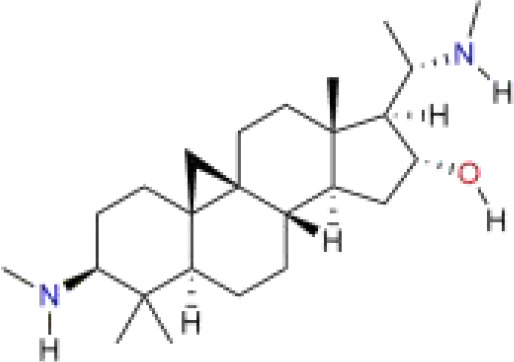	alkaloid	*In vitro*	HepG2 cells, Huh-7 cells.	–	(117)
Schisandrin A	*Schisandra chinensis* (Turcz.) Baill.	C_24_H_32_O_6_	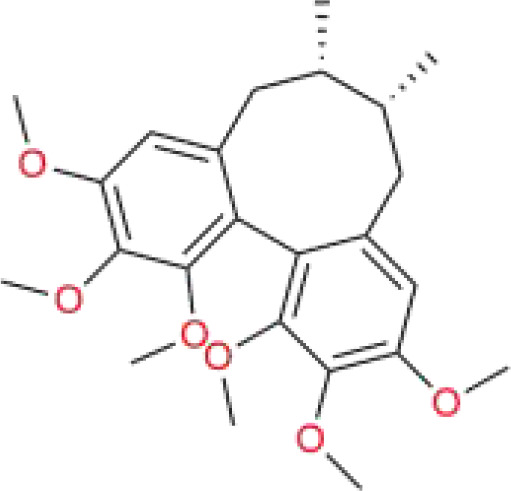	lignan	*In vitro*	Huh-7 cells.	AMPK/mTOR	(118)

Artesunate (ART), as an important derivative of artemisinin, has shown potential advantages in the prevention and treatment of liver cancer. Recently, the team led by Xinyue Liu discovered that ART can induce ferroptosis by regulating iron homeostasis, thereby playing a role in the pathological processes of liver fibrosis and liver cancer (104). To address the issues of poor water solubility and low bioavailability of ART, Dengyun Nie and colleagues have designed a novel nanodelivery system—mesoporous silica nanoparticles (MSN) modified with folic acid (FA) and loaded with ART (MSN-ART-FA). This strategy not only significantly improved the solubility of the drug but also endowed it with tumor-targeting properties. Results from *in vitro* and *in vivo* experiments demonstrate that MSN-ART-FA exhibits significant efficacy in inhibiting the proliferation, migration, invasion, and tumor growth of HCC cells (105). Solamargine (SM) is derived from the plant *Solanum nigrum* Tausch ex Dunal. It inhibits the proliferation of HCC cells by promoting the STAT1/MTCH1 axis in cancer cells, while also inducing apoptosis and ferroptosis (106). Chlorogenic acid (CGA) is abundant in plants such as *Eucommia ulmoides* Oliv. and *Lonicera japonica* Thunb. Numerous studies have shown that CGA can significantly inhibit the proliferation of HCC cells (107). Ling Wu and colleagues found through *in vitro* experiments that CGA can reprogram arachidonic acid metabolism by modulating the PTGS2/AKR1C3/GPX4 signaling pathway, thereby inducing ferroptosis to inhibit the proliferation, migration, and invasion of HCC cells (108). Juglone is a natural naphthoquinone compound, primarily derived from the roots, leaves, fruits, shells, and bark of plants in the Juglandaceae family. Recent studies have shown that juglone activates the FOSL1-HMOX1 axis, which promotes the expression of HO-1 and increases intracellular iron deposition. This subsequently triggers lipid peroxidation and oxidative stress, leading to ferroptosis. As a result, juglone significantly inhibits the proliferation of HCC cells and tumor growth (109). Faberidilactone A is a sesquiterpene dimer extracted from the flower of *Inula japonica* Thunb., and it has shown significant antitumor potential. Recent studies have found that it can exert its anticancer effects through the following mechanisms: First, it activates the apoptotic pathway by inducing mitochondrial dysfunction mediated by ROS, thereby inducing apoptosis. Second, it induces ferroptosis by increasing the consumption of GSH and the accumulation of lipid peroxides, and by inhibiting the activity of GPX4. In addition, it inhibits cell proliferation by causing G2/M cell cycle arrest and suppressing the STAT3 signaling pathway, and it inhibits cell migration by modulating the FAK signaling pathway. *In vivo* experiments have further confirmed that Faberidilactone A effectively inhibits tumor growth and metastasis by suppressing angiogenesis (110). Polyphyllin I (PPI) is a bioactive compound extracted from *Paris polyphylla* Sm. Recent studies have found that PPI significantly inhibits the proliferation, invasion, and metastasis of hepatocellular carcinoma cells by regulating the Nrf2/HO-1/GPX4 axis and inducing ferroptosis and mitochondrial dysfunction (111).

Iberverin is a natural compound extracted from *Brassica oleracea* var. *Capitata* L. Studies have shown that Iberverin can induce apoptosis in HCC cells, inhibit their tumorigenicity, and does not exhibit systemic toxicity (112). Recent research has found that Iberverin can simultaneously downregulate the expression levels of SLC7A11 mRNA and GPX4, thereby inducing lipid peroxidation and ferroptosis, effectively suppressing the proliferation of HCC cells. Moreover, low-dose Iberverin can also enhance the sensitivity of HCC cells to classical ferroptosis inducers (113). Auraptene is a coumarin compound extracted from citrus plants and their fruits. Studies have shown that auraptene can degrade the key ferroptosis defense protein SLC7A11 via the ubiquitin-proteasome pathway, subsequently leading to decreased intracellular levels of GSH and redox imbalance. This ultimately induces ferroptosis in HCC cells, effectively inhibiting the proliferation of HCC cells (114). Ginsenoside RK1 is a saponin compound extracted from *Ginseng* Alph.Wood. Studies have found that Ginsenoside RK1 primarily induces ferroptosis by downregulating the protein and mRNA levels of FSP1, thereby disrupting the cellular redox balance and inhibiting cell proliferation and viability (115). Curcumin is a polyphenolic compound derived from turmeric, which has anti-tumor effects. Studies have found that curcumin can increase the expression of Long-chain acyl coenzyme synthase-4 (ACSL4), leading to an increase in the production of Malondialdehyde (MDA). Additionally, curcumin can reduce the levels of SLC7A11, resulting in decreased production of GSH, which in turn affects the activity of GPX4, ultimately leading to the occurrence of ferroptosis (116). Cyclovirobuxin D (CVB-D) is an alkaloid compound extracted from the roots of *Buxus microphylla* Siebold & Zucc. Studies have shown that CVB-D can significantly increase the intracellular levels of Fe²^+^, MDA, and ROS, while inhibiting the expression of ferroptosis-resistant proteins GPX4 and FSP1. These actions promote ferroptosis and inhibit the growth of HCC cells (117). Schisandrin A (SchA), a lignan with significant biological activity isolated from the fruits of *Schisandra chinensis* (Turcz.) Baill, has been found to exert its anti-hepatocellular carcinoma effects by inducing mitochondrial dysfunction and ferroptosis through the activation of the AMPK/mTOR signaling pathway (118).

## 5 Limitation

In this paper, 645 articles on ferroptosis in HCC were analyzed, and visualization software was used to predict the hotspots and trends of future research. However, there are certain limitations in the course of the study. First of all, the data only came from the English literature in the WoSCC database, ignoring the literature from other databases and important studies in other languages. Second, we included literature from 1 January 2012 to 30 October 2024, and the update of this journal database will also lead to a change in the literature we analyzed, while emerging hotspots after 30 October were not included in the analysis. Finally, using different analysis software can also lead to the omission of some information, resulting in small differences in the results, which does not perfectly present some of the underlying information.

## 6 Conclusion

Based on bibliometrics and visualization methods, this study analyzed the development trend of ferroptosis in HCC. In recent years, the field has developed rapidly, and the output of articles has increased yearly. China, as the most productive country, has the largest number of publications, and the top 10 authors and institutions with the most publications are also from China. The United States has the highest average citation rate and is the most influential country. Hence, strengthening cooperation between China and the United States is conducive to in-depth research in this field. Cell and Nature are the two most frequently cited journals and are authoritative in the field.

Our results show that the study of the pathogenesis and treatment options of ferroptosis in HCC is still a hot topic, in order to find new therapeutic targets. Non-invasive treatment methods such as photodynamic therapy and electrodynamic therapy stand out. Nanomaterials are also a future research trend, with the advantages of high targeting, tumor accumulation, and few side effects, and have great potential in the treatment of HCC. A multitude of studies has found that Chinese medicine molecules can effectively promote ferroptosis in HCC and enhance the sensitivity to sorafenib. When combined with traditional chemotherapy regimens, they can significantly improve the therapeutic efficacy of HCC and reduce drug resistance and side effects. Currently, fundamental research on ferroptosis in HCC is continuously deepening, while clinical trials are relatively scarce. To address the challenges encountered in clinical treatment, future research efforts should place greater emphasis on conducting potential clinical trials and translational studies. During the design phase of basic research, consideration should be given to the potential for translation, employing models and biomarkers that more closely resemble human physiological and pathological conditions to ensure the validity and applicability of research outcomes in clinical settings. Furthermore, enhanced communication and collaboration between basic science researchers and clinical healthcare professionals are essential to facilitate the efficient translation of scientific discoveries into practical clinical treatment plans. Despite the shortcomings of this study, it can still provide research hotspots and trends in this field so as to stimulate new ideas and directions.

## Data Availability

The datasets presented in this study can be found in online repositories. The names of the repository/repositories and accession number(s) can be found in the article/supplementary material.
